# Recalcitrant Perioral Dermatitis Successfully Controlled With Low-Dose Isotretinoin: A Case Report

**DOI:** 10.7759/cureus.105896

**Published:** 2026-03-26

**Authors:** Diego Guarda, Priscilla Marquez, Gerardo Bascuñan, Catalina Montane

**Affiliations:** 1 General Practice, Clínica Andes Salud Puerto Montt, Puerto Montt, CHL; 2 General Medicine, Universidad Andrés Bello, Santiago, CHL; 3 General Practice, Hospital Familiar y Comunitario de Carahue, Carahue, CHL; 4 Dermatology, Universidad de Santiago de Chile, Santiago, CHL

**Keywords:** isotretinoin, ivermectin, low-dose therapy, perioral dermatitis, periorificial dermatitis, refractory treatment, rosacea

## Abstract

We report the case of a 45-year-old woman with recalcitrant perioral dermatitis resistant to multiple topical and systemic therapies who achieved complete remission following treatment with oral isotretinoin. The patient presented with persistent periorificial papules despite ivermectin-based topical regimens, calcineurin inhibitors, compounded anti-inflammatory formulations, and repeated courses of doxycycline. Sustained disease control was achieved with low-dose isotretinoin maintenance therapy. This case highlights the therapeutic challenges associated with refractory perioral dermatitis and supports the potential role of isotretinoin as an antibiotic-sparing strategy in selected patients.

## Introduction

Perioral dermatitis is a chronic inflammatory dermatosis primarily affecting the periorificial regions of the face and is characterized by monomorphic erythematous papules, patches of background erythema, and variable scaling. Although its exact prevalence is uncertain, it is most frequently observed in adult women and may overlap clinically with other inflammatory facial dermatoses, particularly rosacea-spectrum disorders [1,2]. The pathogenesis is considered multifactorial, involving epidermal barrier dysfunction, inflammatory dysregulation, and potential alterations of the cutaneous microbiome [1].

Several triggering factors have been implicated, including topical corticosteroid exposure, cosmetic products, fluorinated compounds, and occlusive environmental conditions. During the COVID-19 pandemic, prolonged mask use has been associated with the exacerbation of periorificial dermatoses and may act as a contributing factor rather than a direct causal mechanism, likely related to mechanical friction, occlusion, and changes in the local microenvironment [3]. Clinically, lesions typically develop gradually rather than presenting as an acute eruption; however, this feature is not specific and should be interpreted in the context of other clinical findings, including lesion distribution, evolution, and exposure history, when distinguishing perioral dermatitis from contact dermatitis or drug-related exanthems [1,2].

The clinical and therapeutic overlap between perioral dermatitis and rosacea has led some authors to propose that both conditions may exist along a shared inflammatory spectrum [4]. This concept is supported by similar responses to anti-inflammatory therapies such as tetracyclines and topical ivermectin, which demonstrate efficacy through immunomodulatory and anti-Demodex mechanisms [4,5]. Although topical agents remain the first-line treatment in mild cases, systemic tetracyclines are widely considered the preferred oral therapy for moderate to severe disease primarily due to their anti-inflammatory and immunomodulatory effects, including the inhibition of neutrophil chemotaxis and modulation of pro-inflammatory cytokines, rather than their antimicrobial activity [2,4]. While tetracyclines are widely used for their anti-inflammatory effects, reports of paradoxical or persistent periorificial dermatitis despite tetracycline therapy are limited and not well characterized in the literature.

Despite these therapeutic options, a subset of patients develops persistent or relapsing disease that fails to respond adequately to conventional treatments, highlighting a therapeutic gap in the management of periorificial dermatitis. In such refractory cases, alternative strategies, including low-dose isotretinoin, have been described, particularly in patients with rosacea-like features or recurrent inflammatory activity. Evidence from rosacea studies suggests that both low-dose (~0.25 mg/kg/day) and very-low-dose regimens may achieve clinical improvement with acceptable tolerability, although relapse after discontinuation is common and optimal maintenance strategies remain unclear [6,7]. However, clear clinical guidelines regarding the escalation of therapy remain limited, further underscoring the need for well-documented clinical observations [2,4].

This case represents a recalcitrant form of perioral dermatitis with a slowly progressive clinical course, overlapping features within the rosacea spectrum, and incomplete responses to multiple conventional therapies. It illustrates the challenges of managing chronic inflammatory periorificial dermatoses in the absence of standardized escalation algorithms and highlights the potential role of isotretinoin as an effective antibiotic-sparing strategy in selected patients with persistent disease.

## Case presentation

**Figure 1:**
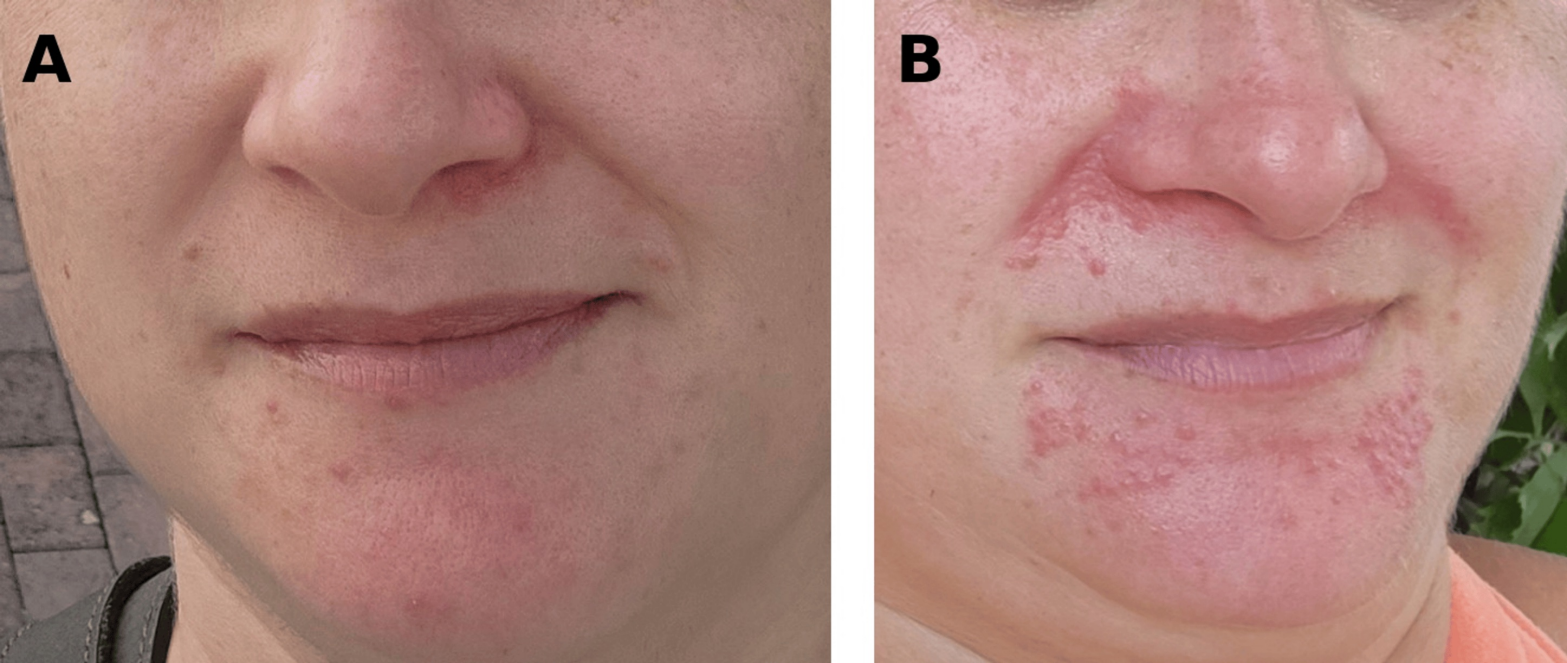
Gradual onset and progressive worsening of periorificial dermatitis (A) Early-stage presentation showing scattered monomorphic erythematous papules localized to the chin and perioral region, with relative sparing of the vermilion border and surrounding facial skin. Lesions appear small, non-pustular, and distributed in a periorificial pattern. (B) Progressive clinical worsening with increased density of erythematous papules coalescing into ill-defined plaques involving the nasolabial folds and perioral area. Background erythema and subtle superficial scaling are visible, consistent with evolving periorificial dermatitis.

A 45-year-old woman presented with a gradually progressive eruption involving the periorificial regions of the face. Her medical history was notable for erythematotelangiectatic rosacea and linear porokeratosis affecting the feet, as well as a family history of rosacea. Approximately four months after prolonged mask use during the COVID-19 pandemic, scattered erythematous papules began to develop insidiously over the chin, nasolabial folds, and periorificial areas while sparing the remainder of the face. Rather than an acute eruption, lesions evolved slowly over time, progressively increasing in number and coalescing into erythematous plaques associated with burning sensation and mild pruritus (Figure 1A, 1B).

The patient denied the use of topical or inhaled corticosteroids.

Perioral dermatitis was diagnosed, and topical ivermectin 1% cream was prescribed nightly for eight weeks along with barrier-repair skincare and the discontinuation of fluorinated products; however, no significant clinical improvement was observed.

Tacrolimus 0.1% ointment was subsequently initiated nightly for eight weeks and was well tolerated but ineffective. A compounded non-comedogenic formulation containing ivermectin 1%, metronidazole 0.75%, alpha-bisabolol 2%, and pimecrolimus 1% was then administered for an additional eight weeks, again without adequate clinical response.

**Figure 2:**
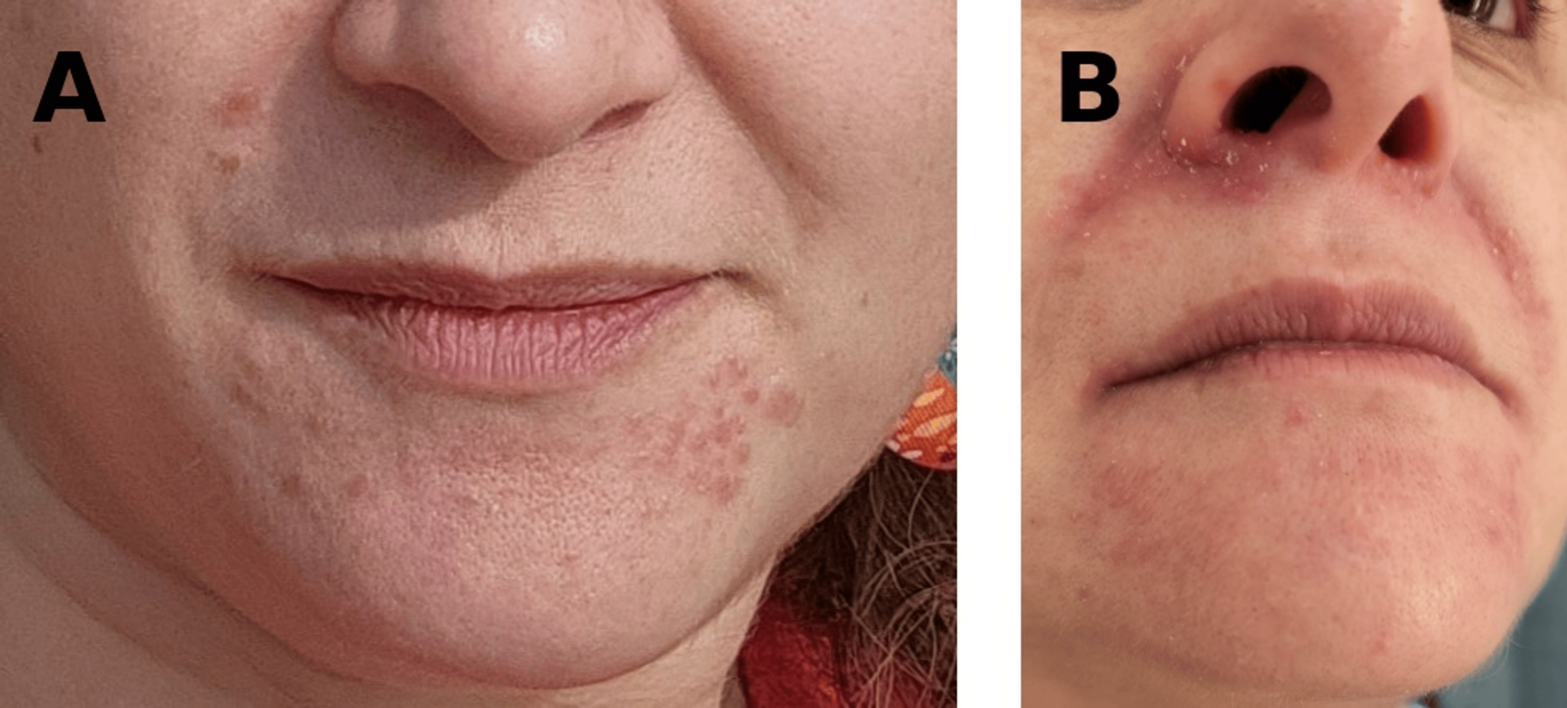
Partial response to doxycycline followed by relapse after treatment discontinuation (A) Partial clinical improvement after anti-inflammatory doxycycline therapy, demonstrating reduction in papule number and decreased erythema, with residual scattered inflammatory lesions over the chin. (B) Disease recurrence after treatment suspension characterized by the reappearance of erythematous papules and patchy periorificial erythema, predominantly along the nasolabial folds.

The first course of doxycycline modified-release 40 mg daily was initiated after mask use had already been discontinued and combined with weekly oral ivermectin pulses (200 µg/kg for two weeks). Partial remission was achieved (Figure 2A), followed by relapse after treatment cessation (Figure 2B).

At least two additional doxycycline courses resulted in transient improvement with recurrent flares.

**Figure 3:**
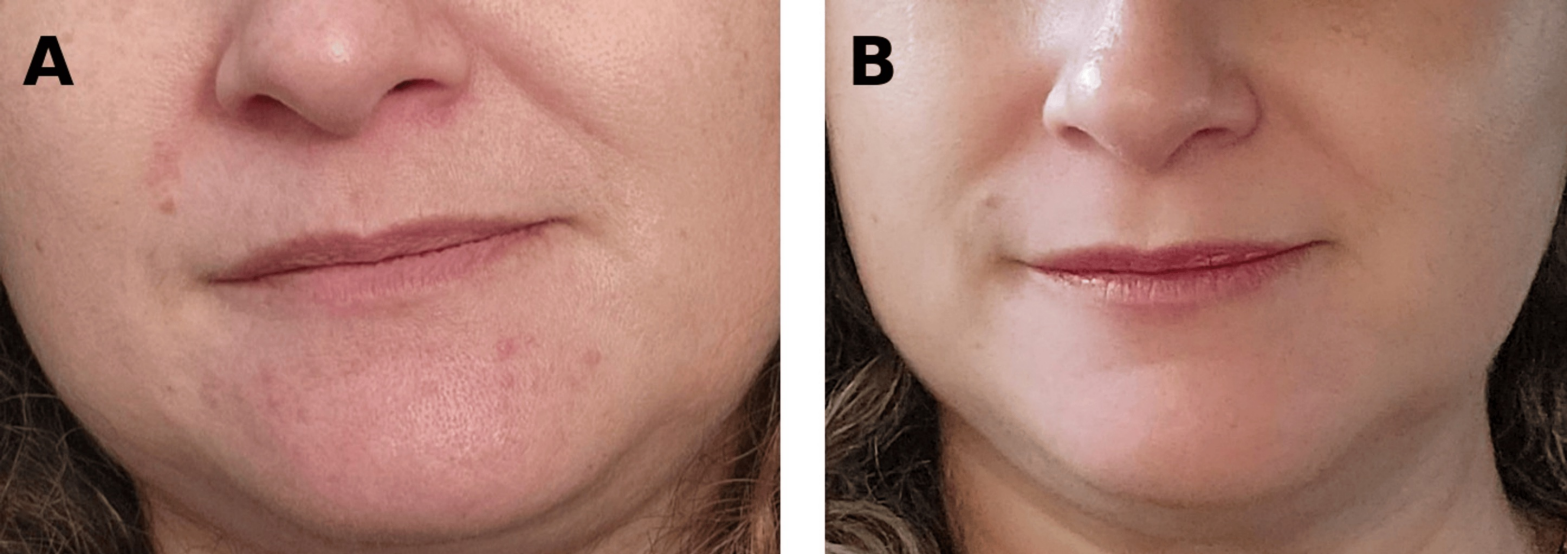
Clinical response to oral isotretinoin therapy (A) Marked reduction in inflammatory papules after the initiation of isotretinoin therapy, with residual faint erythema and minimal scattered perioral lesions, consistent with early therapeutic response. (B) Further improvement under continued isotretinoin exposure demonstrating smoother skin texture and reduction of active inflammatory lesions, though subtle residual erythema remains, compatible with ongoing anti-inflammatory effect.

To minimize prolonged antibiotic exposure, oral isotretinoin therapy was initiated. Baseline laboratory evaluation, including complete blood count, hepatic profile, lipid profile, creatinine, and creatine kinase (CK), was performed prior to treatment initiation, and results were within normal limits. Isotretinoin was started at 20 mg daily, corresponding to a low-dose regimen consistent with doses reported in the literature for rosacea-spectrum inflammatory dermatoses. Patient weight and BMI were not available, representing a limitation in assessing weight-adjusted dosing. Marked clinical improvement was observed at three months (Figure 3A), and therapy was continued for six months alongside barrier-repair measures, achieving near-complete resolution with residual post-inflammatory erythema (Figure 3B).

Laboratory monitoring at one month remained within normal limits, and no further routine hepatic monitoring was performed during the remainder of the treatment course. Baseline and one-month laboratory values are summarized in Table 1.

**Table 1:** Baseline and one-month laboratory evaluation Laboratory evaluation performed prior to isotretinoin initiation and at the one-month follow-up. All parameters remained within normal ranges. Reference values correspond to typical adult laboratory ranges and may vary according to age, sex, laboratory standards, and analytical methods; results should therefore be interpreted within the individual clinical context. AST: aspartate aminotransferase; GOT: glutamic-oxaloacetic transaminase; ALT: alanine aminotransferase; GPT: glutamate-pyruvate transaminase; GGT: gamma-glutamyl transferase; CK: creatine kinase

Test	Baseline	1 month	Unit	Reference range
Hemoglobin	13.5	13.0	g/dL	12-15.5
Leukocytes	6500	5600	/µL	4000-10000
Platelets	255000	230000	/µL	150000-400000
AST (GOT)	25	20	U/L	10-35
ALT (GPT)	20	20	U/L	7-35
GGT	30	25	U/L	9-36
Total cholesterol	190	185	mg/dL	<200
Triglycerides	100	90	mg/dL	<150
Creatinine	0.67	0.70	mg/dL	0.6-1.1
CK	50	65	U/L	30-170

**Figure 4:**
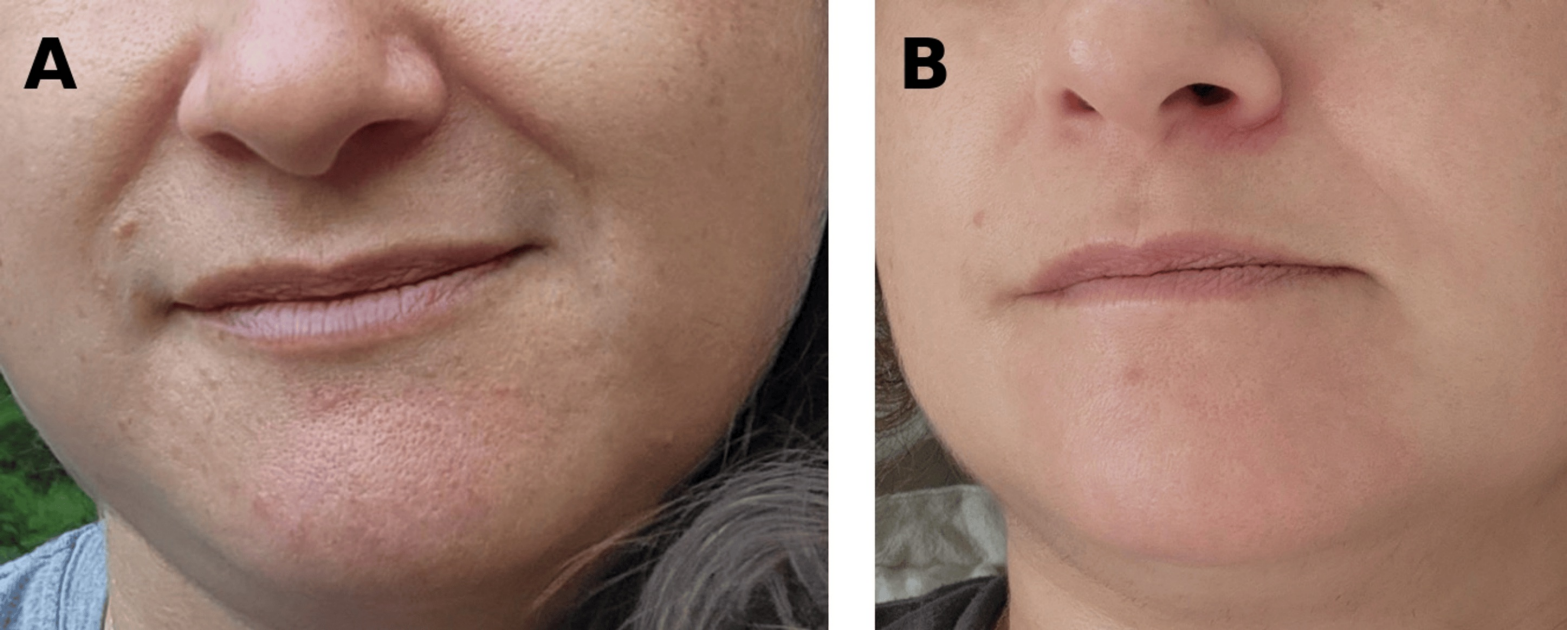
Recurrence after isotretinoin discontinuation and sustained improvement with low-dose maintenance therapy (A) Recurrence of monomorphic erythematous papules and localized periorificial inflammation two months after isotretinoin withdrawal. (B) Marked clinical improvement under low-dose maintenance isotretinoin showing more uniform skin texture and significant reduction of inflammatory lesions, with only a few residual papules and mild background erythema, consistent with controlled but not completely resolved disease activity.

Two months after isotretinoin discontinuation, the patient experienced disease recurrence (Figure 4A). Low-dose anti-inflammatory isotretinoin (5 mg three times weekly) was initiated, resulting in rapid clinical improvement and almost complete clearance of inflammatory lesions within approximately six weeks (Figure 4B).

The sequence of therapeutic interventions and corresponding clinical responses throughout the disease course is summarized in Table 2.

**Table 2:** Therapeutic timeline of treatments and clinical response Chronological overview of therapeutic interventions, treatment duration, and clinical evolution throughout the disease course.

Stage	Treatment	Duration	Clinical context	Response
1	Topical ivermectin 1% + barrier care	8 weeks	Initial diagnosis	No significant response
2	Tacrolimus 0.1% topical	8 weeks	Post-ivermectin failure	No improvement
3	Compounded topical combination	8 weeks	Persistent disease	No response
4	Doxycycline modified-release 40 mg + oral ivermectin	8 weeks	First systemic therapy	Partial remission
5	Additional doxycycline courses	≥2 courses	Recurrent flares	Partial response
6	Isotretinoin 20 mg/day	6 months	Antibiotic-sparing strategy	Complete remission
7	Post-isotretinoin withdrawal period	2 months	Observation after treatment cessation	Clinical relapse
8	Low-dose isotretinoin 5 mg three times weekly	≥6 months follow-up	Anti-inflammatory maintenance therapy	Lesion clearance achieved at ~6 weeks and sustained remission

The patient has remained clinically stable during six months of follow-up under this maintenance regimen, with only residual post-inflammatory erythema and plans for future intense pulsed light (IPL) therapy.

## Discussion

Perioral/periorificial dermatitis typically presents with papules and erythema in perioral, perinasal, or periocular distribution and may involve multifactorial pathophysiology including barrier dysfunction, inflammation, and microbiome alterations [1]. Increasing evidence suggests that disruption of epidermal homeostasis and persistent perifollicular inflammation contribute to the chronic or relapsing course observed in many patients. Although topical corticosteroid exposure is a well-recognized trigger in steroid-induced perioral dermatitis, many individuals present without such a history, representing idiopathic cases that may differ in clinical course and therapeutic response and likely involve alternative inflammatory mechanisms and environmental contributors [2].

In this case, onset after prolonged mask use may reflect occlusion-induced barrier disruption and microenvironmental changes. Reports during the COVID-19 pandemic have described the exacerbation of periorificial dermatoses associated with mask use, supporting a potential role for friction, humidity, and local inflammatory amplification in predisposed individuals [3]. The slowly progressive evolution over several months is consistent with a chronic inflammatory process; however, this finding is not specific and may also be observed in other subacute or chronic conditions and should therefore be interpreted as supportive rather than diagnostic.

Clinical overlap with rosacea remains an important concept. Similarities in morphology, distribution, and treatment response have led some authors to propose that perioral dermatitis may exist along a broader rosacea-like inflammatory spectrum [4]. This framework provides a mechanistic rationale for the use of therapies traditionally employed in rosacea management. Topical ivermectin, for example, has demonstrated efficacy in papulopustular rosacea and exhibits both anti-inflammatory and anti-Demodex activity, mechanisms that may also benefit periorificial inflammatory dermatoses [5]. Although evidence in perioral dermatitis is limited, case reports and small clinical series describe improvement with topical ivermectin in rosacea-like phenotypes [8,9].

Topical calcineurin inhibitors have also been investigated as steroid-sparing options and may be considered as alternative therapies rather than standard first-line treatment, particularly in patients who cannot tolerate or should avoid topical corticosteroids. A randomized controlled trial demonstrated clinical benefit of pimecrolimus 1% in adult perioral dermatitis [10], and additional observational studies support its use when corticosteroids are avoided [11]. Despite these therapeutic approaches, our patient failed to achieve sustained disease control with multiple topical strategies, including calcineurin inhibitors and compounded anti-inflammatory regimens, underscoring the refractory nature of the eruption.

Systemic tetracyclines, particularly anti-inflammatory-dose doxycycline, are considered the first-line oral therapy in moderate to severe perioral dermatitis because of their immunomodulatory effects rather than antimicrobial activity [2,4]. However, in this case, repeated courses produced only partial and transient improvement, with relapse after discontinuation, suggesting that the underlying inflammatory pathways driving disease activity were not fully suppressed by tetracycline therapy alone.

Oral isotretinoin has been proposed as a therapeutic option in difficult-to-treat inflammatory facial dermatoses, including rosacea-like and persistent periorificial dermatitis. Beyond its sebostatic effects, isotretinoin is known to modulate innate immune responses, downregulate Toll-like receptor-mediated inflammatory signaling, reduce neutrophil chemotaxis, and promote the normalization of follicular keratinization. These mechanisms may be particularly relevant in monomorphic papular eruptions characterized by perifollicular inflammation and chronic immune activation. Although direct mechanistic studies in perioral dermatitis remain limited, extrapolation from rosacea research provides a plausible rationale for isotretinoin use through the modulation of the cutaneous microenvironment toward a less inflammatory state; however, this should be interpreted as a hypothesis based on mechanistic and clinical parallels rather than as an established therapy in this condition [6,7].

The clinical course observed in our patient supports this hypothesis. Multiple topical therapies and repeated doxycycline courses failed to induce sustained remission, whereas isotretinoin resulted in the complete clearance of inflammatory lesions. Moreover, rapid clinical improvement during low-dose maintenance therapy and relapse after discontinuation suggest a strong inflammatory component responsive to retinoid-mediated immune modulation. These findings reinforce the concept that selected recalcitrant periorificial dermatitis cases may share pathogenic pathways with rosacea-spectrum inflammatory dermatoses and may benefit from isotretinoin as an antibiotic-sparing strategy.

## Conclusions

This report describes recalcitrant perioral dermatitis resistant to multiple topical and systemic therapies that achieved near-complete remission with oral isotretinoin. Clinical overlap with rosacea and the lack of standardized guidance for refractory disease emphasize the importance of individualized management strategies and antibiotic-sparing approaches. However, these findings are based on a single case, and the use of isotretinoin in perioral dermatitis should be interpreted as a therapeutic hypothesis supported by mechanistic and clinical parallels with rosacea rather than as an established evidence.
